# Organ doses in preterm and full-term neonates and infants — a retrospective study on 1,064 chest radiographs

**DOI:** 10.1007/s00247-022-05324-8

**Published:** 2022-03-18

**Authors:** Birgit Kammer, Karl O. Schneider, Evi Dell’Agnolo, Michael C. Seidenbusch

**Affiliations:** 1 Department of Radiology, University Hospital LMU Munich, Lindwurmstr. 4, 80337 Munich, Germany; 2 Department of Medicine, LMU München, Munich, Germany; 3 Department of Pediatric and Adolescent Medicine, Klinikum Landsberg am Lech, Landsberg am Lech, Germany; 4 BG Klinikum Murnau gGmbH, Murnau, Germany

**Keywords:** Chest, Infant, Neonate, Organ dose, PCXMC phantom, Preterm, Radiation dose, Radiography

## Abstract

**Background:**

Chest radiography is the most frequent X-ray examination performed in the neonatal period. However, commonly used dosimetric entities do not describe the radiation risk sufficiently.

**Objective:**

The aim of this study was to investigate selected organ doses and total body dose of chest radiographs in preterm and full-term neonates and infants.

**Materials and methods:**

In this retrospective study, we evaluated 1,064 chest radiographs of 136 preterm and 305 full-term babies with respect to field size and centering. We calculated the entrance dose from the dose–area product. Upper and lower field borders referred to the corresponding vertebrae. We calculated individual organ doses of the thyroid, the breast, the liver and active bone marrow for each chest radiograph using the neonatal PCXMC program, a Monte Carlo program for calculating patient doses in medical X-ray examinations.

**Results:**

The median field size of chest radiographs ranged from 90 cm^2^ in preterm neonates at birth to 290 cm^2^ in full-term infants at the age of 6 months. Median values of entrance dose varied, depending on age, from 15 μGy to 25 μGy. The median organ doses ranged 1–20 μSv for the thyroid, 3–30 μSv for the breast, 2–20 μSv for the liver and 0.5–3.5 μSv for the bone marrow in preterm and full-term neonates and infants, respectively.

**Conclusion:**

The analysis of chest radiographs in preterm and full-term neonates and infants revealed high variability in field size. By contrast, the entrance dose varied to a minor extent. Organ dose calculations using the PCXMC program might be a valuable tool to calculate the individual radiation risk in neonates and infants.

## Introduction

Radiation protection in neonatal radiology is of utmost importance because of the higher radiation sensitivity of organs and tissues in these children [1]. Previous publications on chest X-rays in preterm and full-term infants exclusively focused on the field size [2], the radiographic technique with ensuing dose [3, 4] or the interrelation of dose, radiographic technique and image quality [5–8]. With the availability of mathematical human phantoms, the concept of the effective dose equivalent became attractive to many research groups to determine organ doses even for medical X-ray exposures [9–13]. In this study, we analyzed the impact of the variation of field size and entrance dose on selected organ doses of chest radiographs in a synthesis using PCXMC, a personal computer (PC)-based Monte Carlo program for calculating patient doses in medical X-ray examinations (Radiation and Nuclear Safety Authority of Finland [STUK], Helsinki) [14].

## Materials and methods

Initially, we retrieved, anonymized and analyzed 1,195 chest radiographs from the picture archiving and communication system. All chest radiographs had been acquired in two neonatal intensive care units of our hospital from Jan. 1, 2009, to Dec. 31, 2010. Chest radiographs of preterm neonates were included not later than 3 months after the calculated due date. Chest images of full-term neonates and infants were evaluated up to 6 months of age. For standardizing the age in preterm and full-term neonates and infants, we calculated the age post-conception for each child. In 112 chest radiographs, the upper and lower field borders could not be delineated; in 11 cases the lateral field borders could not be delineated. In an additional eight cases, two or more field borders were not visible. These cases were excluded, leaving us with 1,064 radiographs for analysis.

The most common referrals for performing chest radiographs were respiratory distress in 26%, assessment of lines and tubes in 17% and infection (sepsis, pneumonia) in 10%. A wide spectrum of indications was presented in approximately 40% of the cases, including persistent ductus arteriosus Botalli, congenital heart disease, lung malformations, anomalies of the diaphragm and other rare diseases. In 7% of cases, acute neonatal emergencies in the delivery room led to insufficient patient history regarding the indication for radiographs. The chest radiographs were acquired with three mobile X-ray machines: one Practix 400 (Philips Medical Systems, Hamburg, Germany) with 30 kW generator power, focus size 0.6; and two Mobilett Plus (Siemens Healthcare, Erlangen, Germany) with 30 kW generator power, focus size 0.8. Radiographic settings were as follows: tube voltage for chest radiographs in preterm neonates with body weights of 450–2,500 g was set at 60 kV. If the neonate reached the due date (full-term), the tube voltage ranged from 60 kV to 65 kV. The current–time product (mAs) was chosen from exposure tables based on neonate’s/infant’s age and weight and was in the range of 0.8–2.5 mAs. The body weight was known in 811 patients and was corrected in the missing patients according to the growth charts of Fenton and Kim [15].

**Fig. 1:**
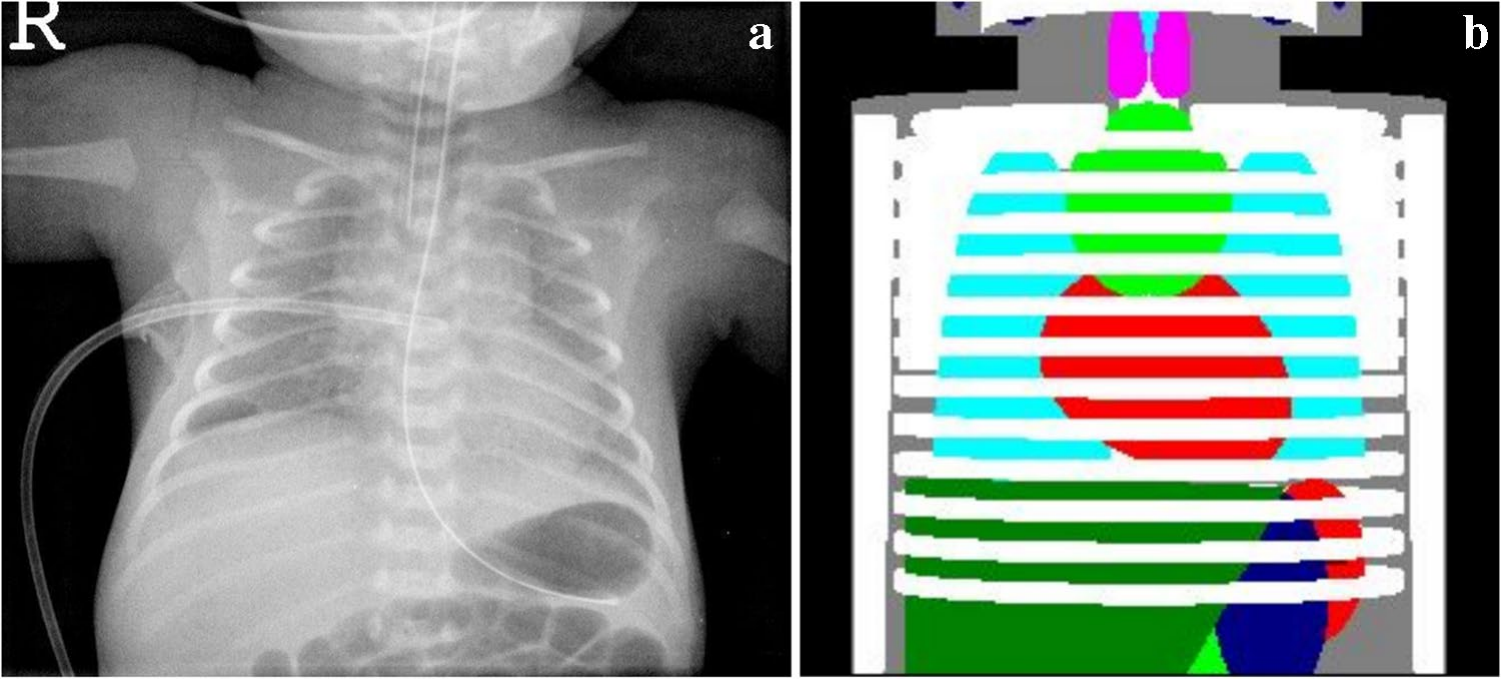
Calculation of organ doses on an anteroposterior (AP) supine chest radiograph performed on a 1-day-old in a preterm girl (30th gestational week) based on the age-matched corresponding MIRD (Radiation and Nuclear Safety Authority of Finland, Helsinki) phantom image of the PCXMC program, a Monte Carlo program for calculating patient doses in medical X-ray examinations. **a** AP radiograph. **b** Dose calculation. *Dark blue* stomach, *Dark green* liver, *Light blue* lungs, *Light green* thymus, *Pink* thyroid*, Red* heart and spleen, *White* skeleton*, Gray* soft tissues

All X-ray exposures were performed with additional filtration of 1.0 mm aluminum plus 0.1 mm copper, and with a focus-detector distance of 100 cm. All parameters were in accordance with the guidelines of the European Commission [16] and the German Federal Medical Association [17]. All chest radiographs were obtained in anteroposterior projection in supine position with the image plates in direct contact with the patients. The dose–area product was measured with sensitive diamentors (PTW, Freiburg, Germany). The chest radiographs were acquired digitally using computed radiographic image plates and read out with various processing devices (ADC Compact Plus and 2 Solo; Agfa, Mortsel, Belgium). To calculate individual organ doses for each neonate and infant from the dose–area product values measured during each exposure by Monte Carlo simulations, we used the PCXMC algorithm. To do so, we transferred the X-ray field of the chest radiograph of each child to a mathematical neonate-size PCXMC phantom [14], which we adjusted according to the neonate’s/infant’s individual body weight and length (Fig. 1). Exposure parameters and radiation doses depend on patient’s somatic properties. As the relative physical development of a single patient in a mixed collective of preterm and full-term babies cannot be clearly described by the patient’s age post-partum, the age post conception was chosen. We used Sigma-Plot 10.0 (Systat Software Inc., San Jose, CA) to create figures and charts.

We obtained local ethics committee approval for this retrospective study. The study was an analysis of anonymized digital images, so no objections were raised.

## Results

We included 1,064 chest images of 441 neonates and infants during their stay in the neonatal intensive care units, 179 females and 262 males, in the study. One hundred twenty-five of 136 (92%) of the premature babies were neonates at time of imaging (Table 1). Only 11 (8%) received the chest films after the newborn period. Table 2 illustrates the age of the full-term babies when they received their chest radiographs. As shown 104 of 305 (34%) of the full-term babies received their chest radiographs in the neonatal period.

**Table 1:** Distribution of the number of premature neonates and infants according to post conception age and the day of chest imaging

Post-conceptional age (weeks)	Number of patients					Total
	0–7 days	7–14 days	14–21 days	21–28 days	>28 days	
24–28	15	0	0	0	0	15
28–32	37	1	2	1	1	42
32–36	38	0	2	3	7	50
36–40	22	2	2	0	3	29
Total	112	3	6	4	11	136

**Table 2:** Distribution of the number of full-term neonates and infants according to post conception age and the day of chest imaging

Post-conceptional age (weeks)	Number of patients					Total
	0–7 days	7–14 days	14–21 days	21–28 days	>28 days	
40–44	71	14	9	10	3	107
44–48	0	0	0	3	40	43
48–52	0	0	0	0	34	34
52–56	0	0	0	0	48	48
56–60	0	0	0	0	35	35
60–64	0	0	0	0	38	38
Total	71	14	9	13	198	305

Fifty-two female preterm girls received 223 chest radiographs; 127 full-term girls received 251 chest radiographs. Among boys, 254 chest radiographs were performed in 84 preterm boys and 336 chest radiographs in 178 full-term boys.

Most neonates and infants, 338 (77%), had only one or two chest radiographs; 87 (20%) neonates and infants had between 3 and 9, and 15 (3%) neonates and infants had 10 or more than 10 chest radiographs. In one female preterm infant, 37 chest radiographs were acquired within the first 2 postpartum months.

**Fig. 2:**
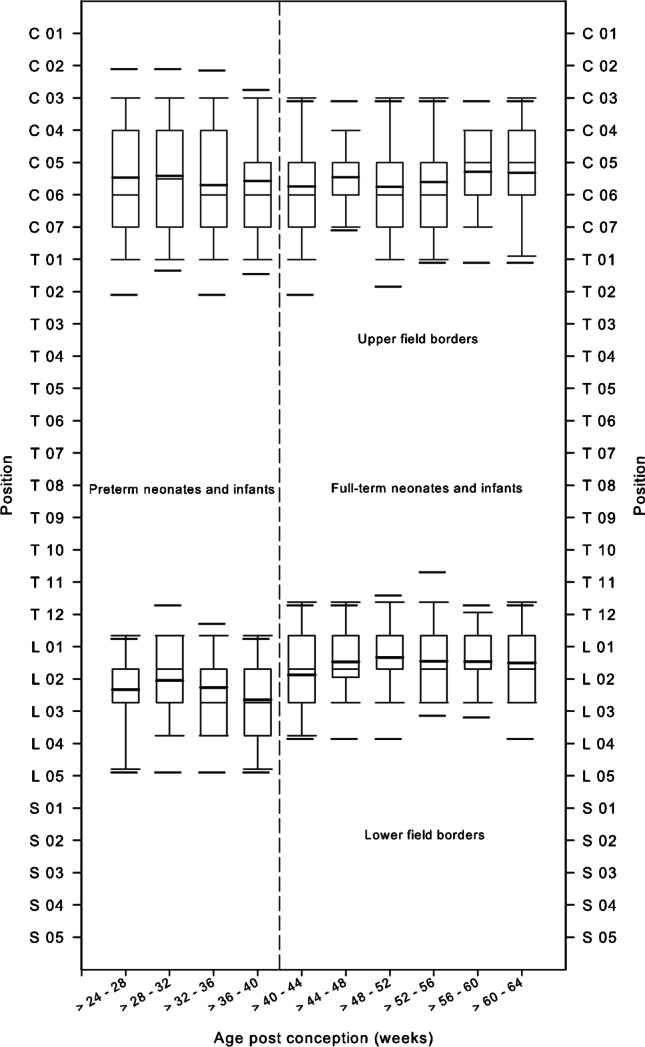
The upper and lower field borders of chest radiographs in premature and full-term neonates and infants are illustrated by means of box and whisker plot relative to the vertebrae in the longitudinal axis in relation to the post-conception age. Thick bars in the boxes are median values, thin bars in the boxes are mean values, box extremes are the 10th and the 90th percentile values, the thick black bars outside the boxes are the 5th and the 95th percentiles

**Fig. 3:**
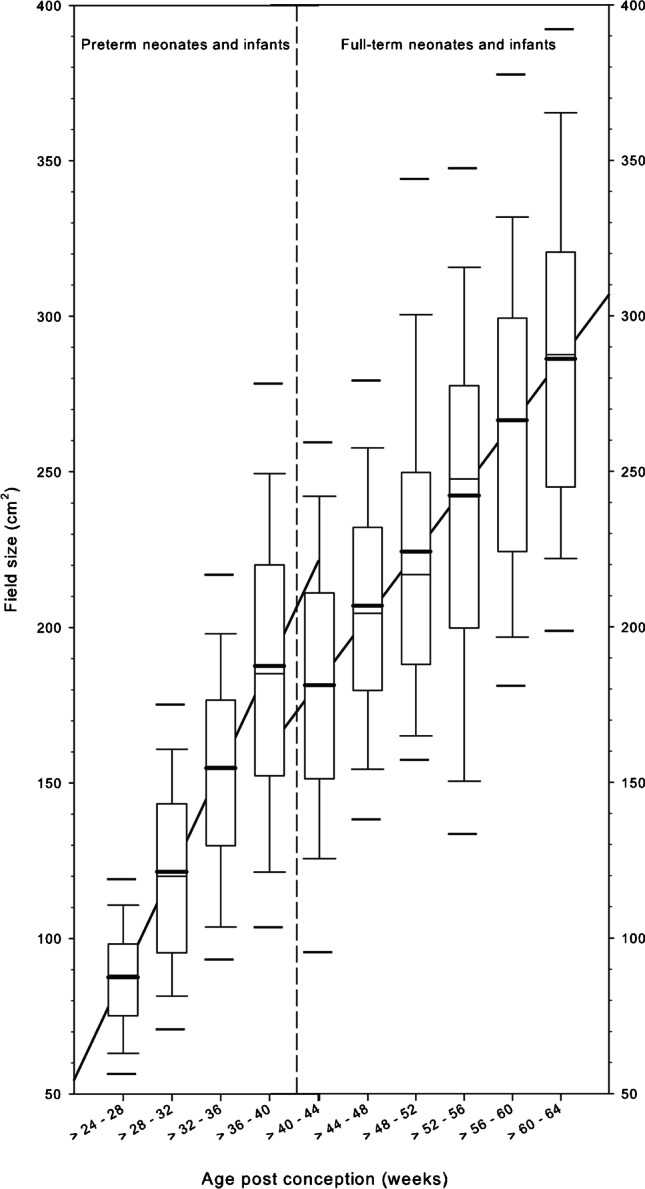
The field sizes of chest radiographs of premature and full-term neonates and infants are illustrated by means of box and whisker plot in relation to the post-conception age (weeks). Thick bars in the boxes are median values, thin bars in the boxes are mean values, box extremes are the 10th and the 90th percentile values, the thick black bars outside the boxes are the 5th and the 95th percentiles

In 761 (71%) chest radiographs, the upper field border was at the tip of the mandible; in 298 (28%) cases, it was at the level of the upper thoracic aperture (T1/T2). In the remaining five (0.5%) cases, collimation was so poor that the maxillae were exposed. Concerning the lateral collimation, the proximal thirds of the humeri were exposed in 753 (71%) cases. However, more than the proximal third of the humeri or the entire upper extremity was irradiated in 309 (29%) cases. Figure 2 illustrates the variability of the upper and lower field borders depending on the age, divided for preterm and full-term neonates and infants. The median values of the upper field borders were between C4 and C6. The median values for the lower field border were between L1 and L2. The field sizes of chest radiographs increased with age in preterm and full-term neonates and infants (Fig. 3). The smallest field size was 35 cm^2^ in a preterm neonate, and the largest was 475 cm^2^ in a 6-month-old full-term infant. The median field size of preterm neonates was in the range of 90 cm^2^ at birth and increased to 290 cm^2^ in full-term infants at the age of 6 months.

**Fig. 4:**
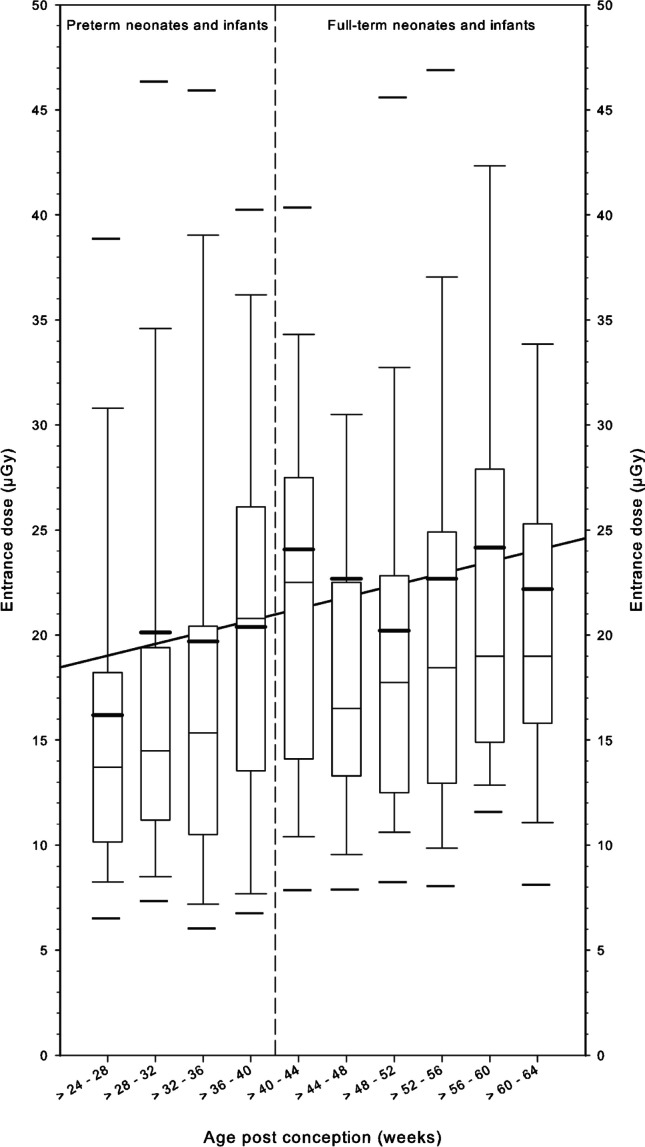
Entrance dose of premature and full-term neonates and infants is illustrated by means of box and whisker plot in relation to the post-conception age (weeks). Thick bars in the boxes are median values, thin bars in the boxes are mean values, box extremes are the 10th and the 90th percentile values, the thick black bars outside the boxes are the 5th and the 95th percentiles

Median values of entrance doses were in the range of 15–20 μGy in preterm neonates and infants and close to 25 μGy in full-term neonates and infants (Fig. 4). The 10th percentiles of preterm and full-term neonates/infants were in the range of 5–10 μGy. The 90th percentiles in both groups ranged 30–45 μGy.

**Fig. 5:**
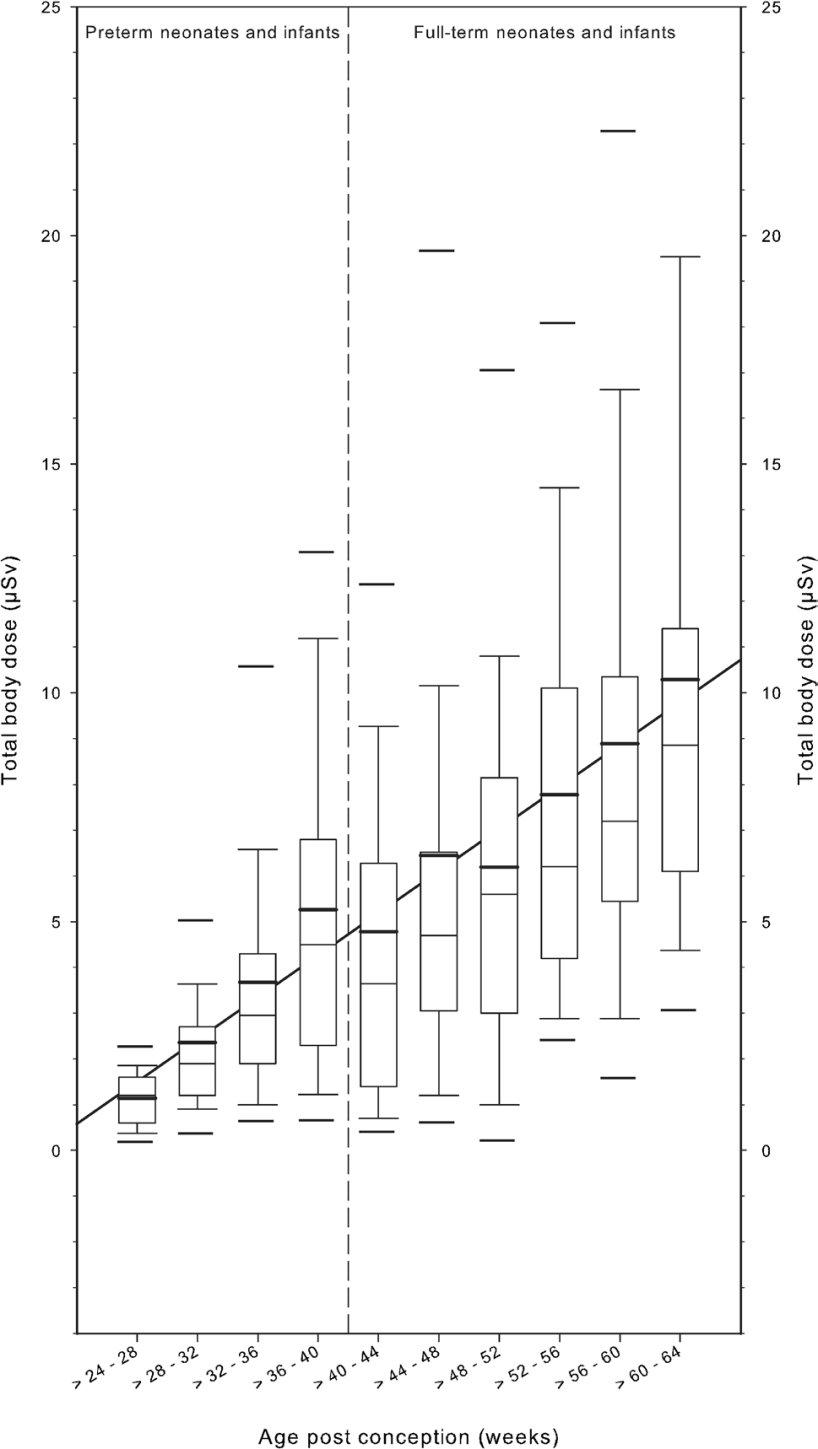
Total body dose is depicted by means of box and whisker plot for premature and full-term neonates and infants in relation to the post-conception age (weeks). Thick bars in the boxes are median values, thin bars in the boxes are mean values, box extremes are the 10th and the 90th percentile values, the thick black bars outside the boxes are the 5th and the 95th percentiles

Following the critical arguments of Drexler et al. [18], we do not favor the effective dose. To regard the scattered dose at the field edges, we calculated the total body dose provided by the PCXMC program. The median values of the total body dose (Fig. 5) of preterm neonates and infants were in the range of 1–4 μSv and in full-term neonates and infants, 5–10 μSv.

**Fig. 6:**
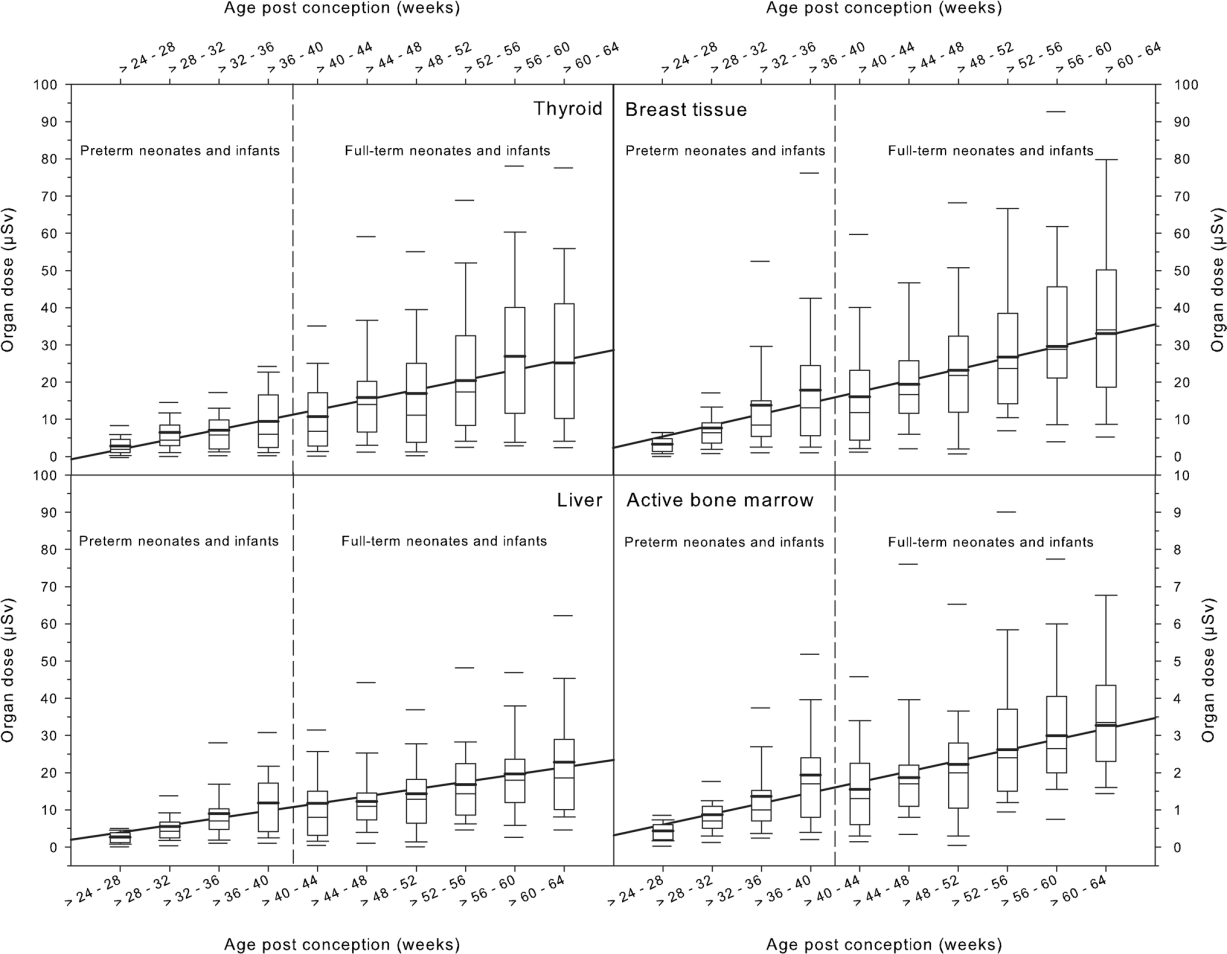
Organ doses of the thyroid, breast tissue, liver and active bone marrow are visualized by means of box and whisker plots for premature and full-term neonates and infants in relation to the post-conception age (weeks). Thick bars in the boxes are median values, thin bars in the boxes are mean values, box extremes are the 10th and the 90th percentile values, the thick black bars outside the boxes are the 5th and the 95th percentiles

**Fig. 7:**
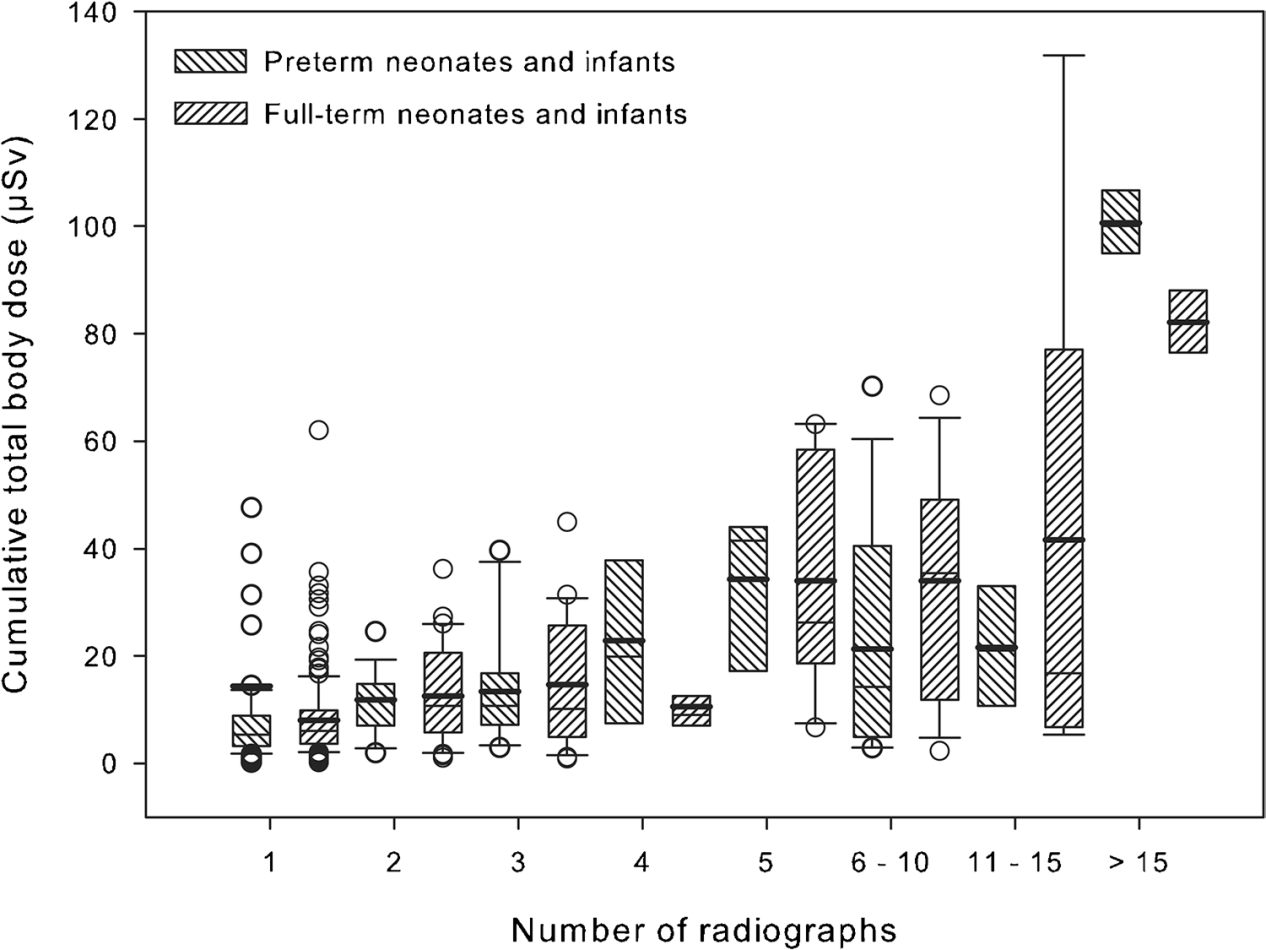
Total body doses are displayed by means of box and whisker plot for premature and full-term neonates in relation to the number of chest radiographs. Thick bars in the boxes are median values, thin bars in the boxes are mean values, box extremes are the 10th and the 90th percentile values, the thick black bars outside the boxes are the 5th and the 95th percentiles Circles are outliers. These are larger premature neonates, i.e. more than 36th gestational weeks and full-term neonates with higher birth weights

Figure 6 shows the organ doses of the four selected organs in preterm and full-term neonates and infants. The median values of the thyroid organ dose were in the range of 1–20 μSv, the median values of the organ doses to the breast varied 3–30 μSv, the median values of the organ doses to the liver were 2–20 μSv, and the median values of the organ doses to the bone marrow were 0.5–3.5 μSv, depending on post-conception age. The lines drawn for visual support of linear extrapolations illustrate that the values of organ doses of preterm neonates and infants are clearly below those of full-term neonates and infants. Figure 7 shows the cumulative total body dose in relation to the number of radiographs.

## Discussion

Many national and international guidelines on radiographic images in pediatrics have been published within the last 25 years by national authorities and international commissions [16, 17, 19]. These recommendations refer to image quality criteria and good radiographic technique and provide dose reference levels. Because chest radiographs are the most common radiographic examinations in neonates, infants and young children [20], and the radiation risk in this age group is higher than in older children and adults [1, 21], it is of great importance to choose the most appropriate dose indicator. Until now, no consensus has existed among neonatologists, medical physicists, and pediatric radiologists as to which dosimetric quantity defines the radiation risk most appropriately. Research groups have used the dose–area product, the entrance air kerma, the entrance surface dose or the entrance dose [3, 8, 10, 12, 16, 22–24] to describe radiation exposure. Other authors have preferred the effective dose [10, 21, 25, 26] to calculate the radiation risk using conversion factors provided by Rosenstein et al. [27]. Smans et al. [28] used the PCXMC program as well as the anthropomorphic voxel baby phantom [11] to perform Monte Carlo simulations to calculate organ doses.

The size of the X-ray field basically determines the imparted energy to the patient. This has been measured in several publications [4, 12, 23]. In our study, the median value of the field size of the chest radiographs in preterm neonates and infants ranged from 85 cm^2^ to 175 cm^2^ dependent on post-conception age. Similar values were reported by Puch-Kapst et al. [26]. The median value of the field size of the chest radiographs of full-term neonates at birth in our study was 170 cm^2^, which is in the same range as that reported by two large surveys [12, 22]. However, two departments in Gunn et al.’s [23] multicenter study reported considerably higher mean radiation fields of between 220 cm^2^ and 320 cm^2^ for chest radiographs obtained in the newborn period. By adjusting the field size to the normalized PCXMC neonate phantom for the infant’s individual body size and weight, we reconstructed the individual organ doses of all chest radiographs considering the specific exposure conditions of each neonate/infant, a fact that might make this study unique. In our survey the field size of 75% of the chest radiographs was in the optimal range as defined in the European guidelines, indicating that it was correctly centered. The lower field edges of 25% of all full-term newborns were below L2. This means that the radiation field was slightly oversized. Lowe et al. [6] described that in five neonatal care units, between 50% and 90% of chest radiographs exceeded the optimal field size. Smans et al. [28] found a variation of field size up to 100% in follow-up chest radiographs. Donadieu et al. [25] did not use the individual radiation field to calculate the individual organ doses. In contrast, Wilson-Costello et al. [21] calculated organ dose based on “standard” and “modified” field settings. They found differences in organ doses by a factor of three when comparing the two field settings. One study investigated the increase of the radiation field in the horizontal axis of newborn babies to assess the unnecessary exposure of the upper extremities [29]; unfortunately, the active bone marrow dose was not estimated in that study.

In our study the mean entrance dose values of chest radiographs in preterm babies from birth to the 6th postnatal month were in the range of 15 μGy in preterm babies to close to 20 μGy in full-term infants. Several research groups reviewed the literature of the entrance dose of chest radiographs in infants and found a wide range of the mean entrance doses of 6–160 μGy [8, 12]. Puch-Kapst et al. [26] reported entrance dose values of 15 μGy in very-low-birth-weight preterm babies. However, these dose values were not measured during routine imaging, but rather were obtained by free in-air kerma measurements under idealized radiographic conditions. Thus, the daily occurring variation of the radiographic parameters was not represented in their survey. Generally speaking, all departments that have reported low entrance doses for chest radiographs in infants have adhered to the recommendations of the European guidelines concerning the radiographic technique, e.g., film-focus distance, kilovoltage and additional filtration [16]. On the other hand, the departments that have had higher entrance dose values for chest radiographs in infants have mostly used low kilovoltages in the range of 45–55 kV [4, 8, 12, 30]. Furthermore, two large dosimetric surveys in very low birth weight preterm infants did not mention or even omitted additional filtration [25, 30]. In other studies, the film-focus distance was significantly below 80 cm [22, 30, 31] or not explicitly mentioned [25].

If we compare published organ doses of other reports with our study, interestingly the organ doses that are especially relevant in chest radiographs in newborns, i.e. breast, thyroid, liver and bone marrow, were not reported by all research groups. Not surprisingly, the organ dose values varied widely between publications. The cumulative organ doses reported by Donadieu et al. [25] could not be compared with our study because their proportion of chest radiographs was only 6–10%. The calculation of the median effective dose for different X-ray examinations renders it impossible to identify the relative contribution of poor radiographic technique [21, 25, 26]. Datz et al. [30] computed organ doses of tightly collimated chest radiographs with those of no collimation at all. The thyroid dose was five times higher than in our study, despite exact collimation and some adjustments of good radiographic technique recommended by the European guidelines [16]. Unfortunately, neither the breast dose nor the liver dose was reported in the study by Datz et al. In contrast, Sharma et al. [31] reported organ dose measurements for 38 neonatal chest radiographs, also using the PCXMC phantom. The thyroid dose was in the same range as in our survey. However, the breast dose was five times higher and the liver dose seven times higher than in our study. These significant differences can only be explained by the five times higher median entrance dose caused by the low kilovoltage and the avoidance of additional filtration. The relatively low thyroid dose and the higher liver dose can be explained by the low centering of the chest radiographs. Consequently, the thyroid was not directly irradiated, but on the other hand more liver tissue was exposed to radiation. Finally, the bone marrow dose with the PCXMC is significantly underestimated. This also applies to our study. Wilson-Costello et al. [21] indicated that only 50% of the hematopoiesis of preterm infants was found in the bones, mainly the skull and the long bones. However, 40% of the hematopoietic cells are in the liver and the remainder in the spleen and kidneys [32]. Therefore, the calculation of the active bone marrow dose is greatly underestimated with the PCXMC program and the voxel phantom. This is especially relevant for chest radiographs within the first 6 months of age.

Most medical physicists and epidemiologists prefer the calculation of the effective dose as a more powerful dosimetric quantity for assessing radiation risk. However, we are convinced that the computation of selected organ doses is more useful than the calculations of the cumulative effective dose for defining radiation risk. It might be suitable for radiologic examinations of large body parts with many organs being irradiated, e.g., babygrams or abdominal series. This applies to three surveys on preterm newborn babies [21, 25, 26]. The great majority of the radiologic examinations, 85%, were babygrams in Donadieu’s survey [25]. In Wilson-Costello’s study this proportion was 40% [21], and it was only 30% in the work of Puch-Kapst et al. [26]. Because newborn neonates are small, even slight deviations in the X-ray field of chest radiographs can increase the doses to neighboring organs. Incorrect collimation (+4 cm) or false centering of chest radiographs at the lower field edge led to a 50% higher liver dose [33]. Furthermore, inappropriately high entrance dose can increase the breast and thyroid dose despite tight collimation. Finally, the most relevant drawback of the use of effective dose in neonates and infants is the fact that weighting factors calculated for adults have been used, and these simply cannot be transferred to the higher radiosensitivity organs of early life. The median values of calculated total body dose increased by a factor of three when more than 5 chest radiographs were obtained. However, when more than 15 chest radiographs were acquired, the median total body dose increased by a factor of ten.

## Conclusion

Our retrospective study and analysis of a large number of papers indicate that the hitherto routinely used dosimetric quantities, like entrance dose, dose–area product/air kerma product, entrance surface dose and effective dose are of limited value to sufficiently describe the assessment of radiation risk in neonates/infants. Consequently, they are not useful to define reference dose levels. In contrast, determining the organ doses of organs that are particularly sensitive to radiation, such as the thyroid, breast, liver, and red bone marrow, allows for a much more meaningful analyses of radiation exposure.

Therefore, we suggest the development of a preterm infant PCXMC phantom, in addition to the neonatal phantom, that considers the larger size liver with its active bone marrow dose.

In the future the assessment of organ doses might be easily achievable by using refined age-appropriate anthropomorphic phantoms in combination with automatic image recognition by artificial intelligence and the routinely used radiographic technique [34].
